# Supplementary material for: The early-life exposome modulates the effect of polymorphic inversions on DNA methylation
Source: Commun Biol. 2022 May 12;5:455. doi: 10.1038/s42003-022-03380-2 (PMC9098634; doi:10.1038/s42003-022-03380-2)
Supplement: Supplementary file 2 — Supplementary Information [file 42003_2022_3380_MOESM2_ESM.pdf]

The early-life exposome modulates the effect of polymorphic inversions on DNA methylation

Natàlia Carreras-Gallo, Alejandro Cáceres, Laura Balagué-Dobón, Carlos Ruiz-Arenas, Sandra Andrusaityte, Ángel Carracedo, Maribel Casas, Leda Chatzi, Regina Grazuleviciene, Kristine Bjerve Gutzkow, Johanna Lepeule, Léa Maitre, Mark Nieuwenhuijsen, Remy Slama, Nikos Stratakis, Cathrine Thomsen, José Urquiza, John Wright, Tiffany Yang, Geòrgia Escaramís, Mariona Bustamante, Martine Vrijheid, Luis A Pérez-Jurado, Juan R González

Supplementary material

Figures

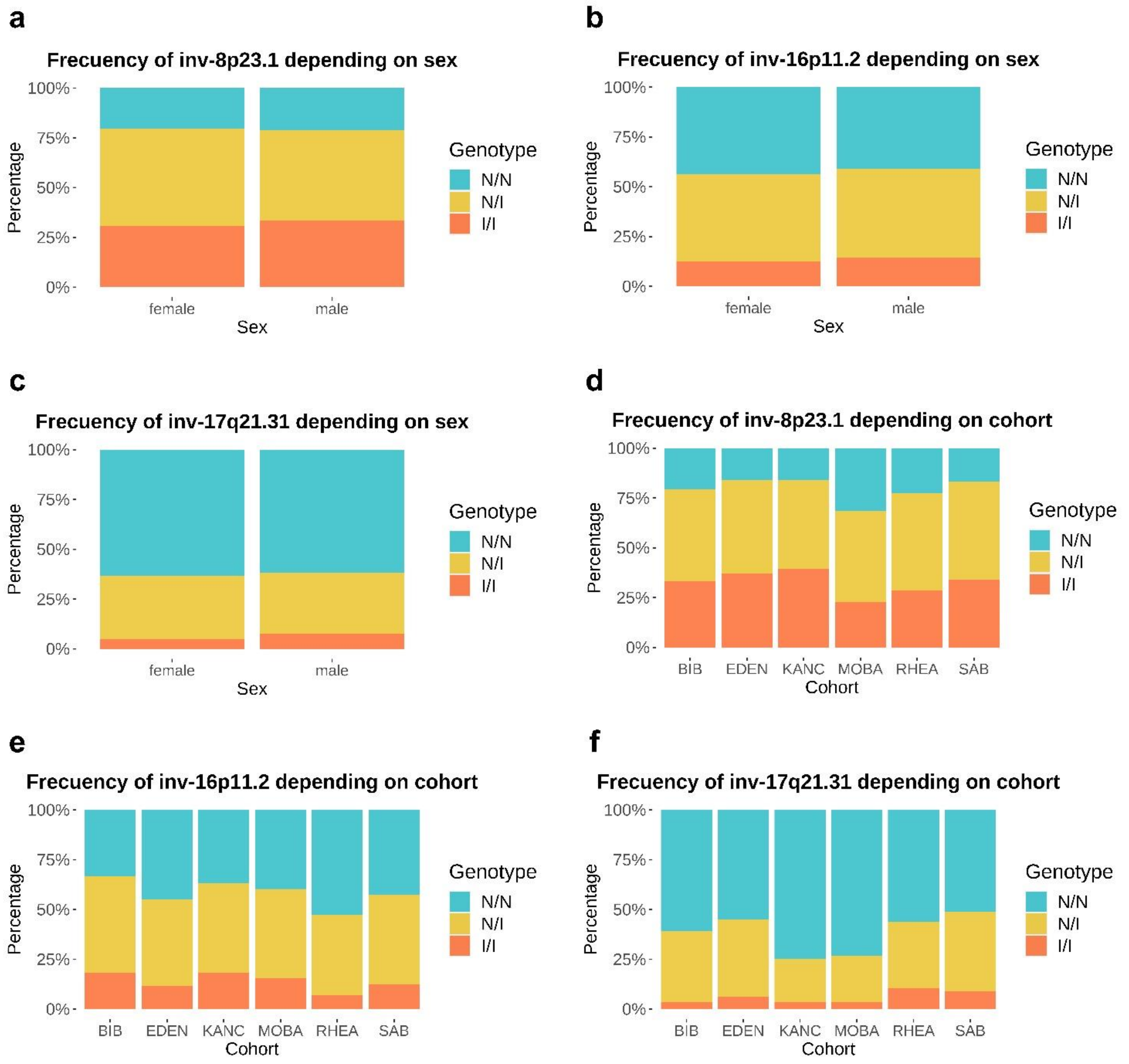

Supplementary Figure 1 | Genotype inversion frequencies for inv-8p23.1, inv-16p11.2, and inv-17q21.31 depending on sex (a-c) and cohort (d-f). In blue, non-inverted/non-inverted (N/N) individuals: in yellow, non-inverted/inverted (N/I) individuals; in orange, inverted/inverted (I/I) individuals.

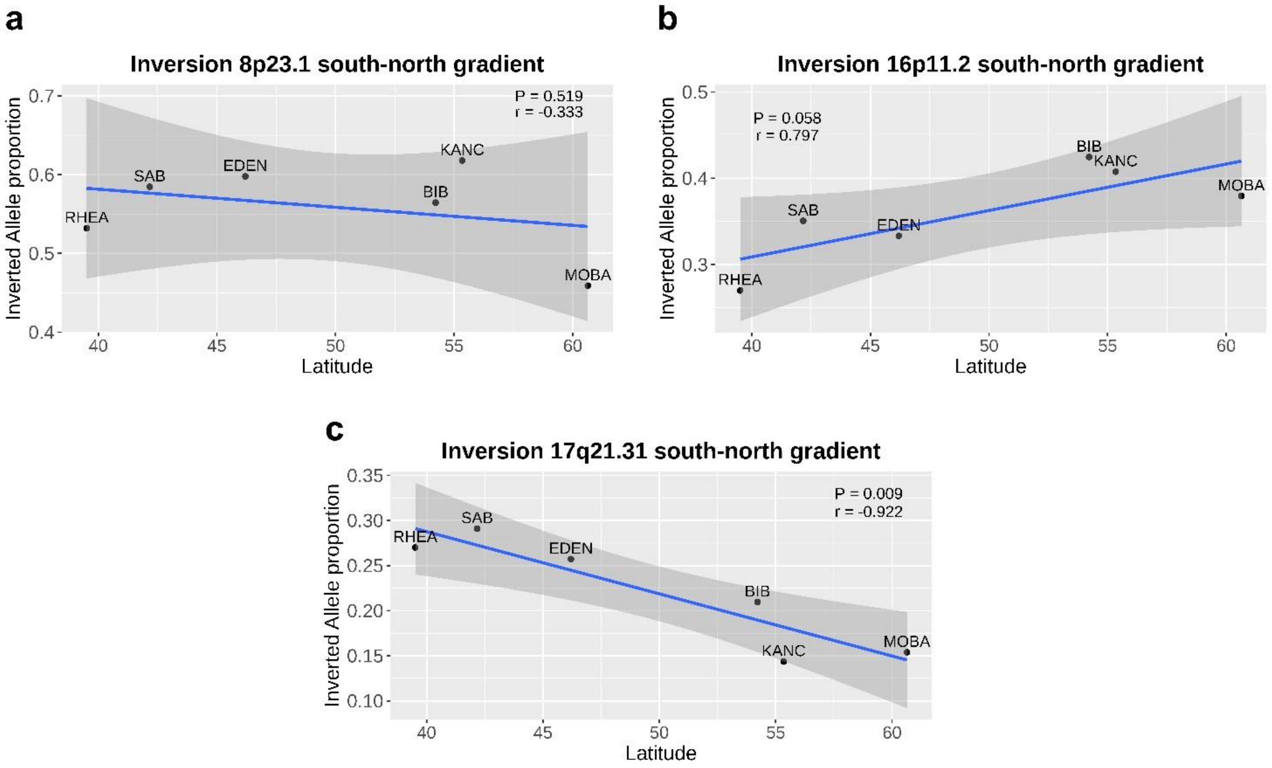

Supplementary Figure 2 | South-north gradient inv-8p23.1, inv-16p11.2, and inv-17q21.31. The x-axis shows the latitude of each cohort in degrees. The y-axis shows the frequency of the inverted allele (from 0 to 1). The p-value and the correlation are annotated in the plot. The grey shading represents the 95% confidence level interval for predictions from a linear model.

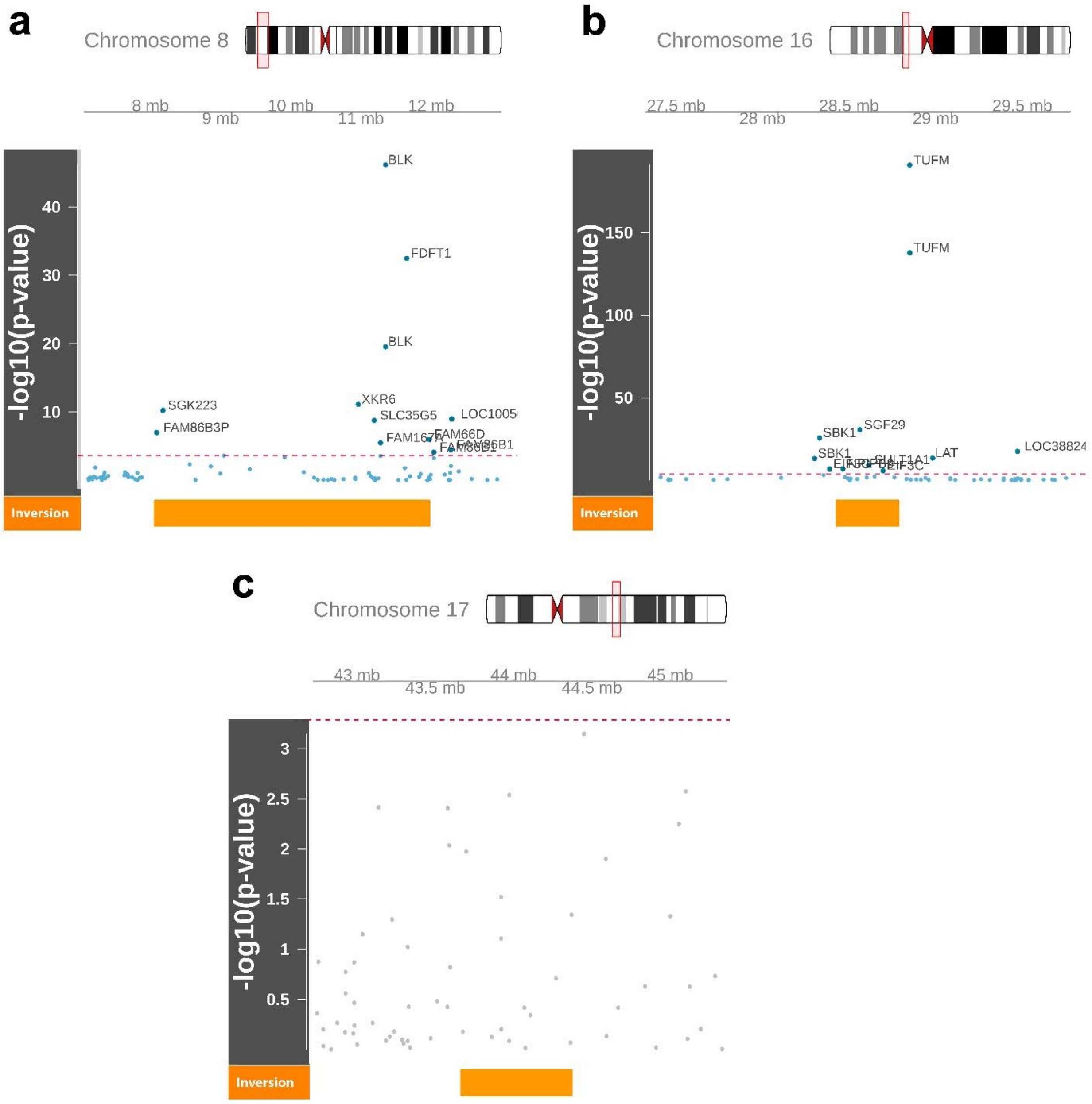

Supplementary Figure 3 | Inversion status as expression quantitative trait loci (eQTL) of multiple genes within and surrounding the inversion region. a) Inv-8p23.1; b) Inv-16p11.2; c) Inv-17q21.31. The x-axis shows the chromosome position (from 1Mb before the left breakpoint of the inversion region to 1Mb after the right breakpoint). The y-axis shows the $-\log_{10}$ (p-value) from the meta-analysis of the association between the differential expression of the genes and the inversion genotypes. The dashed red line indicates Bonferroni's threshold of significance. Green points are genes with significant associations and those in grey are non-significant. The orange block illustrates the inversion region.

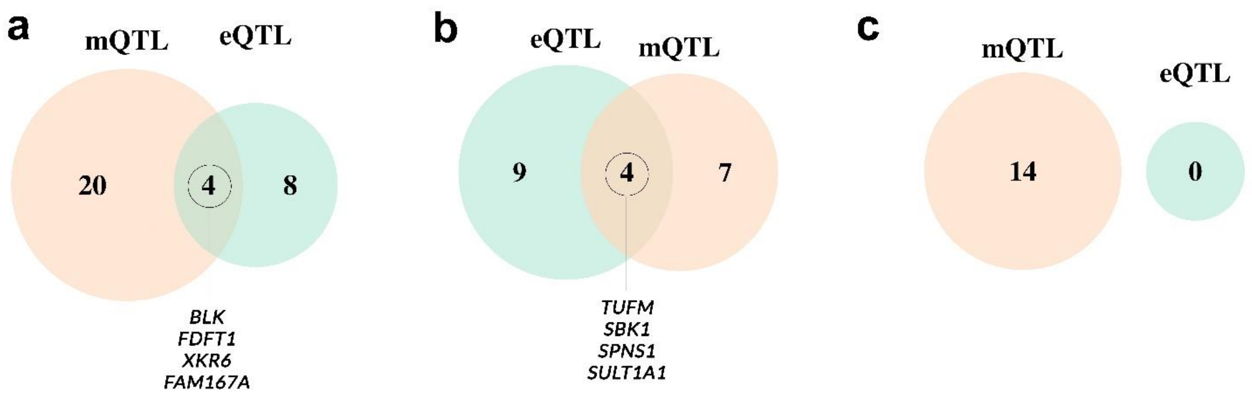

Supplementary Figure 4 | Venn diagram comparing the genes differentially expressed (eQTL) and methylated (mQTL) according to the inversion haplotype. a) Inv-8p23.1; b) Inv-16p11.2; c) Inv-17q21.31. The genes that overlapped are annotated with their gene symbol.

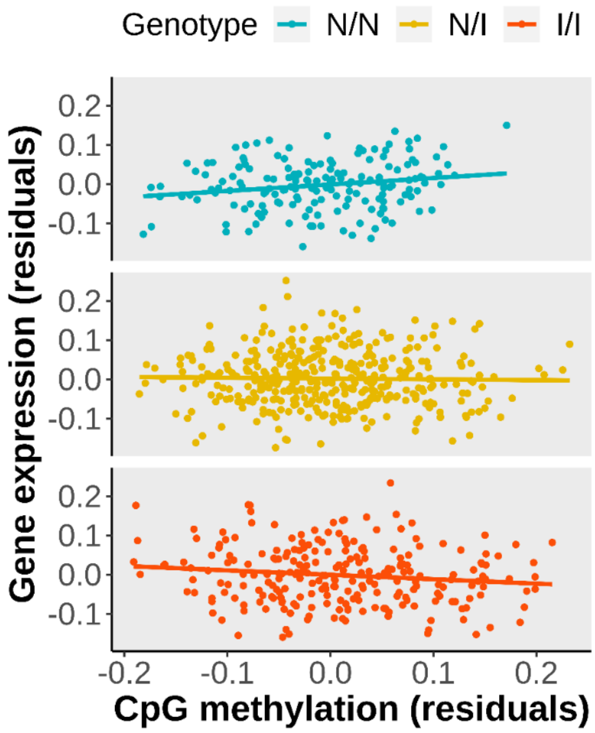

Supplementary Figure 5 | Relationship between the expression and the CpG methylation of *TDH* gene stratified by inv-8p23.1 genotype. Blue points and lines illustrate non-inverted homozygous (N/N), yellow illustrate heterozygous (N/I), and orange illustrate inverted homozygous (I/I) individuals for inv-8p23.1.

Tables

Supplementary Table 1 | Characteristics of the participants from the HELIX project contributing to the analysis of gene expression.

			Overall (N=790)
**Sex**		**NK_6**	
female	355 (44.9%)	Mean (SD)	0.034 (0.039)
male	435 (55.1%)	Range	0.000 - 0.294
**Age**		**Bcell_6**	
Mean (SD)	7.881 (1.465)	Mean (SD)	0.116 (0.038)
Range	5.437 - 11.984	Range	0.021 - 0.257
**Cohort**		**CD4T_6**	
BIB	78 (9.9%)	Mean (SD)	0.196 (0.063)
EDEN	78 (9.9%)	Range	0.031 - 0.449
KANC	142 (18.0%)	**CD8T_6**	
MOBA	179 (22.7%)	Mean (SD)	0.131 (0.048)
RHEA	148 (18.7%)	Range	0.001 - 0.496
SAB	165 (20.9%)	**Gran_6**	
**Inversion at 8p23.1**		Mean (SD)	0.442 (0.111)
N/N	167 (21.1%)	Range	0.019 - 0.752
N/I	385 (48.7%)	**Mono_6**	
I/I	238 (30.1%)	Mean (SD)	0.086 (0.027)
**Inversion at 16p11.2**		Range	0.000 - 0.202
N/N	338 (42.8%)		
N/I	349 (44.2%)		
I/I	103 (13.0%)		
**Inversion at 17q21.31**			
N/N	497 (62.9%)		
N/I	245 (31.0%)		
I/I	48 (6.1%)		

Supplementary Table 2 | Characteristics of the participants from the HELIX project contributing to the analysis of DNA methylation.

	Overall (N=1009)
**Sex**	
female	457 (45.3%)
male	552 (54.7%)
**Age**	
Mean (SD)	7.941 (1.566)
Range	5.437 - 11.984
**Cohort**	
BIB	93 (9.2%)
EDEN	138 (13.7%)
KANC	195 (19.3%)
MOBA	195 (19.3%)
RHEA	187 (18.5%)
SAB	201 (19.9%)
**Inversion at 8p23.1**	
N/N	209 (20.7%)
N/I	476 (47.2%)
I/I	324 (32.1%)
**Inversion at 16p11.2**	
N/N	428 (42.4%)
N/I	442 (43.8%)
I/I	139 (13.8%)
**Inversion at 17q21.31**	
N/N	628 (62.2%)
N/I	318 (31.5%)
I/I	63 (6.2%)
**NK_6**	
Mean (SD)	0.034 (0.040)
Range	-0.000 - 0.294
**Bcell_6**	
Mean (SD)	0.114 (0.037)
Range	0.021 - 0.257
**CD4T_6**	
Mean (SD)	0.194 (0.063)
Range	0.019 - 0.477
**CD8T_6**	
Mean (SD)	0.129 (0.047)
Range	0.001 - 0.496
**Gran_6**	
Mean (SD)	0.448 (0.110)
Range	0.019 - 0.806
**Mono_6**	
Mean (SD)	0.086 (0.027)
Range	0.000 - 0.231

Supplementary Table 3 | Characteristics of the genomic inversions genotyped by *scoreInvHap*.

scoreInvHap Inversion	Cytogenetic Location	Length Inversion (bp)	CpGs Inversion region	CpGs Inversion region +/- 1Mb
**inv1_004**	1p22.1	774	0	365
**inv1_008**	1q31.3	1198	1	187
**inv2_002**	2p22.3	718	0	121
**inv2_013**	2q22.1	4254	0	71
**inv3_003**	3q26.1	2279	0	6
**inv6_002**	6p21.33	873	0	4084
**inv6_006**	6q23.1	4120	0	148
**inv7_003**	7p14.3	5254	0	250
**inv7_005**	7p11.2	73939	2	174
**inv7_011**	7q11.22	12694	0	134
**inv7_014**	7q36.1	2077	0	909
**inv8_001**	8p23.1	3924860	786	848
**inv11_004**	11p12	1383	0	1337
**inv11_001**	11q13.2	4748	0	39
**inv12_004**	12q13.11	19286	0	277
**inv12_006**	12q21.2	1032	0	201
**inv14_005**	14q23.3	861	0	276
**inv16_009**	16p11.2	364169	63	401
**inv17_007**	17q21.31	710890	109	666
**inv21_005**	21q21.3	1058	0	77
**invX_006**	Xq13.2	90847	0	0

Supplementary Table 4 | Characteristics of the fetuses from interrupted pregnancies contributing to the analysis of DNA methylation.

	Overall (N=39)
**Sex**	
female	19 (48.7%)
male	20 (51.3%)
**Inversion at 8p23.1**	
N/N	7 (17.9%)
N/I	18 (46.2%)
I/I	14 (35.9%)
**Inversion at 16p11.2**	
N/N	17 (43.6%)
N/I	19 (48.7%)
I/I	3 (7.7%)
**Inversion at 17q21.31**	
N/N	16 (41.0%)
N/I	22 (56.4%)
I/I	1 (2.6%)
