# Supplementary material for: The early-life exposome modulates the effect of polymorphic inversions on DNA methylation
Source: Commun Biol. 2022 May 12;5:455. doi: 10.1038/s42003-022-03380-2 (PMC9098634; doi:10.1038/s42003-022-03380-2)
Supplement: Supplementary file 3 — Description of Additional Supplementary Files [file 42003_2022_3380_MOESM3_ESM.pdf]

Description of Additional Supplementary Files

File name: Supplementary Data 1
Description: Differentially expressed genes according to the inversion status for inv-8p23.1, 16p11.2, and 17q21.31. The table illustrates the significant results (p-value adjusted < 0.05). TE: Estimate of treatment effect; seTE: Standard error of treatment estimate; lower: Lower limit of confidence interval; upper: Upper limit of confidence interval; DIR BIB: sign of the β value in Born in Bradford cohort; DIR EDEN: sign of the β value in Etude des Déterminants pré et postnatals du développement et de la santé de l'Enfant cohort; DIR KANC: sign of the β value in Kaunas cohort; DIR MOBA: sign of the β value in Norwegian Mother and Child cohort; DIR RHEA: sign of the β value in Rhea cohort; DIR SAB: sign of the β value in Infancia y Medio Ambiente cohort; p.adj: P-value with Bonferroni's correction.

File name: Supplementary Data 2
Description: Differentially methylated CpG sites according to the inversion status for inv-8p23.1, inv16p11.2, and inv-17q21.31. The table illustrates the significant results (p-value adjusted < 0.05). The gene symbol and gene group from each CpG site is annotated based on the Illumina HM450 manifest file (version 1.2).TE: Estimate of treatment effect; seTE: Standard error of treatment estimate; lower: Lower limit of confidence interval; upper: Upper limit of confidence interval; DIR BIB: sign of the β value in Born in Bradford cohort; DIR EDEN: sign of the β value in Etude des Déterminants pré et postnatals du développement et de la santé de l'Enfant cohort; DIR KANC: sign of the β value in Kaunas cohort; DIR MOBA: sign of the β value in Norwegian Mother and Child cohort; DIR RHEA: sign of the β value in Rhea cohort; DIR Page 7 of 20 SAB: sign of the β value in Infancia y Medio Ambiente cohort; p.adj: P-value with Bonferroni's correction.

File name: Supplementary Data 3
Description: Validation of differentially methylated CpG sites according to the inversion status for inv-8p23.1, inv-16p11.2, and inv-17q21.31 in heart tissue from fetuses from interrupted pregnancies. The table illustrates the significant results with a p-value < 0.005. The gene symbol from each CpG site is annotated based on the Illumina HM450 manifest file (version 1.2). logFC: estimate of the log2-fold-change corresponding to the effect or contrast; AveExpr: average log2-expression for the probe over all arrays and channels; t: moderated tstatistic; B: log-odds that the gene is differentially expressed; p.adj: P-value with Bonferroni's correction.

File name: Supplementary Data 4
Description: List of exposures assessed in the analysis from the HELIX project. The exposures are grouped in 12 families and in two periods (postnatal and pregnancy).

File name: Supplementary Data 5
Description: Differentially methylated CpG sites according to the inversion – exposure interaction across 3 inversions (inv-8p23.1, 16p11.2, and 17q21.31) and 64 exposures in early-life. The table illustrates the significant results (p-value adjusted < 0.05). The DIR BIB, DIR EDEN, DIR KANC, DIR MOBA, DIR RHEA, and DIR SAB represents the sign of the β value in each of the cohorts when the analysis was performed independently. The gene symbol and gene group from each CpG site is annotated based on the Illumina HM450 manifest file (version 1.2). TE: Estimate of treatment effect; seTE: Standard error of treatment estimate; lower: Lower limit of confidence interval; upper: Upper limit of confidence interval; DIR BIB: sign of the β value in Born in Bradford cohort; DIR EDEN: sign of the β value in Etude des Déterminants pré et Page 8 of 20 postnatals du développement et de la

santé de l’Enfant cohort; DIR KANC: sign of the β value in Kaunas cohort; DIR MOBA: sign of the β value in Norwegian Mother and Child cohort; DIR RHEA: sign of the β value in Rhea cohort; DIR SAB: sign of the β value in Infancia y Medio Ambiente cohort; p.adj: P-value with Bonferroni's correction.

File name: Supplementary Data 6
Description: Summary of the Table S5 counting the number of significant interactions where an exposure takes action. The “Inversion interactions” specifies the inversion/s that interact with the exposure.

File name: Supplementary Data 7
Description: Source data underlying Fig 2a, 3a, and 3e
