# Supplementary material for: The early-life exposome modulates the effect of polymorphic inversions on DNA methylation
Source: Commun Biol. 2022 May 12;5:455. doi: 10.1038/s42003-022-03380-2 (PMC9098634; doi:10.1038/s42003-022-03380-2)
Supplement: Supplementary file 11 — Reporting Summary [file 42003_2022_3380_MOESM11_ESM.pdf]

nature portfolio

Corresponding author(s): Juan Ramón González

Last updated by author(s): Apr 6, 2022

Reporting Summary

Nature Portfolio wishes to improve the reproducibility of the work that we publish. This form provides structure for consistency and transparency in reporting. For further information on Nature Portfolio policies, see our Editorial Policies and the Editorial Policy Checklist.

Statistics

For all statistical analyses, confirm that the following items are present in the figure legend, table legend, main text, or Methods section.

n/a	Confirmed	
☐	☒	The exact sample size (n) for each experimental group/condition, given as a discrete number and unit of measurement
☐	☒	A statement on whether measurements were taken from distinct samples or whether the same sample was measured repeatedly
☐	☒	The statistical test(s) used AND whether they are one- or two-sided<br>*Only common tests should be described solely by name; describe more complex techniques in the Methods section.*
☐	☒	A description of all covariates tested
☐	☒	A description of any assumptions or corrections, such as tests of normality and adjustment for multiple comparisons
☐	☒	A full description of the statistical parameters including central tendency (e.g. means) or other basic estimates (e.g. regression coefficient) AND variation (e.g. standard deviation) or associated estimates of uncertainty (e.g. confidence intervals)
☐	☒	For null hypothesis testing, the test statistic (e.g. F, t, r) with confidence intervals, effect sizes, degrees of freedom and P value noted<br>*Give P values as exact values whenever suitable.*
☒	☐	For Bayesian analysis, information on the choice of priors and Markov chain Monte Carlo settings
☐	☒	For hierarchical and complex designs, identification of the appropriate level for tests and full reporting of outcomes
☐	☒	Estimates of effect sizes (e.g. Cohen's d, Pearson's r), indicating how they were calculated

Our web collection on statistics for biologists contains articles on many of the points above.

Software and code

Policy information about availability of computer code

Data collection	Any custom code or software used in our analysis is available at DOI: 10.5281/zenodo.6417926 (URL: https://zenodo.org/badge/latestdoi/296552532).
Data analysis	Any custom code or software used in our analysis is available at DOI: 10.5281/zenodo.6417926 (URL: https://zenodo.org/badge/latestdoi/296552532).

For manuscripts utilizing custom algorithms or software that are central to the research but not yet described in published literature, software must be made available to editors and reviewers. We strongly encourage code deposition in a community repository (e.g. GitHub). See the Nature Portfolio guidelines for submitting code & software for further information.

Data

Policy information about availability of data

All manuscripts must include a data availability statement. This statement should provide the following information, where applicable:

- Accession codes, unique identifiers, or web links for publicly available datasets
- A description of any restrictions on data availability
- For clinical datasets or third party data, please ensure that the statement adheres to our policy

The HELIX data warehouse has been established as an accessible resource for collaborative research involving researchers external to the project. Access to HELIX data is based on approval by the HELIX Project Executive Committee and by the individual cohorts. Further details on the content of the data warehouse (data catalog) and procedures for external access are described on the project website (http://www.projecthelix.eu/index.php/es/data-inventory). The data used in this analysis are not available for replication because specific approvals from HELIX Project Executive Committee and the University of Southern California Institutional Review Board must be obtained to access them.

March 2021

Field-specific reporting

Please select the one below that is the best fit for your research. If you are not sure, read the appropriate sections before making your selection.

☒ Life sciences ☐ Behavioural & social sciences ☐ Ecological, evolutionary & environmental sciences

For a reference copy of the document with all sections, see nature.com/documents/nr-reporting-summary-flat.pdf

Life sciences study design

All studies must disclose on these points even when the disclosure is negative.

Sample size	We analyzed data from the Human Early Life Exposome (HELIX) project, a multicenter European cohort (Spain, UK, France, Lithuania, Norway, and Greece). From 1,301 children, we included 1,009 children in the analysis.
Data exclusions	As pre-stablished, samples with discordant sex, duplicated, high heterozygosity, and relatives were filtered out. We also removed the non-European samples and the individuals without methylation or genetic data.
Replication	We used an independent study (prenatal heart tissue) to confirm the effect of the three common inversions in methylation.
Randomization	HELIX is a multicenter study of 6 cohorts, within each cohort, molecular data was obtained. As explained in the manuscript, we adjusted for all the necessary covariates.
Blinding	Non applicable.

Reporting for specific materials, systems and methods

We require information from authors about some types of materials, experimental systems and methods used in many studies. Here, indicate whether each material, system or method listed is relevant to your study. If you are not sure if a list item applies to your research, read the appropriate section before selecting a response.

Materials & experimental systems

n/a	Involved in the study	
☒	☐	Antibodies
☒	☐	Eukaryotic cell lines
☒	☐	Palaeontology and archaeology
☒	☐	Animals and other organisms
☐	☒	Human research participants
☒	☐	Clinical data
☒	☐	Dual use research of concern

Methods

n/a	Involved in the study	
☒	☐	ChIP-seq
☒	☐	Flow cytometry
☒	☐	MRI-based neuroimaging

Human research participants

Policy information about studies involving human research participants

Population characteristics	We included in our analysis sex, age, population stratification, and cell type as covariates.
Recruitment	This study is based in the multicenter HELIX, that has been described elsewhere.
Ethics oversight	All studies received approval from the ethics committees of the centers involved and written informed consent was obtained from all participants.

Note that full information on the approval of the study protocol must also be provided in the manuscript.
